# Russian Norms for 500 General-Knowledge Questions

**DOI:** 10.3389/fpsyg.2020.545304

**Published:** 2020-09-30

**Authors:** Beatriz Martín-Luengo, Oksana Zinchenko, Maria Alekseeva, Yury Shtyrov

**Affiliations:** ^1^Center for Cognition and Decision Making, Institute of Cognitive Neuroscience, National Research University - Higher School of Economics, Russian Federation, Moscow, Russia; ^2^Department of Clinical Medicine, Center of Functionally Integrative Neuroscience (CFIN), Aarhus University, Aarhus, Denmark

**Keywords:** general knowledge questions, recognition, calibration curves, metacognition, Russian language

General knowledge varies from one country to another; therefore, the mere translation of knowledge-based experimental tools from one language to another is usually not enough. This is one of the conclusions that can be extracted from the results of the Program for International Student Assessment (PISA), a measure of the knowledge achieved by 15-year-olds. This assessment provides each participating country (79 in 2018) with a comparative measure of the efficiency of their educational programs within an internationally agreed common framework and allows them to identify the most effective educational practices as well as gaps in curricula. Over the years, this periodic measure has reflected clear differences between countries in different areas of knowledge. The PISA program assesses mathematics, sciences, and reading, designed as an indicator of “how well the students master key subjects in order to be prepared for real-life situations in the adult world” (PISA, 2018). An example of a question used in the PISA test is: “As a meteoroid approaches Earth and its atmosphere, it speeds up. Why does this happen?: (1) The meteoroid is pulled in by the rotation of Earth; (2) The meteoroid is pushed by the light of the Sun; (3) The meteoroid is attracted to the mass of Earth; (4) The meteoroid is repelled by the vacuum of space.” The PISA program was first conducted in 2000 and despite the educational changes implemented by each government to increase student competitiveness, significant differences between countries remain. These and similar data suggest that general knowledge varies from country to country owing, among others, to the variety of educational practices in combination with the access to the information, cultural practices, etc.

In cognitive research, different materials are used such us pictures, words, sentences, texts, etc. Several studies have shown that the mere translation of semantic materials into the native language of each country is not sufficiently adequate even for simple items such as words. Akinina et al. (2015) ran a study aimed primarily at validating semantic and visual material (words and pictures) in Russian to ultimately be used in experiments for designing clinical interventions of language recovery. They found that name agreement scores for visual stimuli influence the latencies in both object and action naming. They also found that name agreement and frequency were the dimensions with the highest variability depending on the use of the word in a specific area. Similar results have also been found in Spanish (Cuetos and Alija, 2003), Japanese (Nishimoto et al., 2012), and other languages. To conclude, experimental materials such as words should be normativized or at least carefully selected from language databases to avoid any distortion of results. This highlights a similar, if not more acute need for more complex materials such as general-knowledge questions (GKQs).

Traditionally, the classical reference for GKQs is the seminal study conducted by Nelson and Narens (1980). However, their study was carried out with a population from the USA, and despite the authors' effort to avoid cultural references and therefore enable a wider use, the PISA reports still show us that the accuracy values can differ across countries. Recently, Tauber et al. (2013) conducted a study to update the results of Nelson and Narens in which possible differences among US states were also considered and controlled in terms of accuracy and other measures. Along the same lines, Duñabeitia et al. (2016) ran a normative study of GKQs in Spain to provide a cross-cultural validation of Tauber's data with a Spanish population. This research illustrates the need for similar normative studies in each country and languages, which should be culturally and linguistically specific.

The aim of the present study is to validate in the Russian language a large pool of GKQs on different topics that can be used in different areas of study from Psychology to Neuroscience within the Russian Federation (RF). Russian is one of the most spoken languages in the world, the largest native language in Europe, one of the six official languages of the UN, and the second most used languages on the Internet globally^1^. Yet, there are still no normative general-language questions for this language, the gap we set out to fill in the current study. We decided to use multiple-choice questions because they can be used in a broad range of experiments, provide straightforward approach to quantifying response accuracy, and are particularly suitable for use in surveys or experiments in which the time of stimulus presentation is controlled and/or limited. Moreover, multiple-choice question tests are widely used to measure general knowledge (e.g., PISA tests, GRE, etc), but their difficulty often relies on the foils presented along with the correct answer. The accuracy of responses to the question: “What is the name of the so-called powerhouse of the cell?” will dramatically diverge if the four alternatives offered are “mitochondria, ribosome, Golgi apparatus or vesicle” vs. “purpurin, mitochondria, DNA, feet”. Because the accuracy can vary depending on the alternatives included, it is not an easy task to adequately transform a free recall task to multiple-choice questions, while the other way around is easier. Moreover, multiple-choice questions are widely used in experimental, clinical, and neuroscience research (Luna et al., 2011; Arnold et al., 2013; Higham, 2013; Chua et al., 2017; Griffiths and Higham, 2018; Mangels et al., 2018; Martín-Luengo et al., 2018; Navajas et al., 2018; Williams et al., 2018; Coane and Umanath, 2019). Therefore, we decided to validate multiple-choice questions and provide the percentage of each alternative selected along with their corresponding confidence ratings.

Metacognitive evaluations such as retrospective confidence judgments provide valuable information about the selection of a specific answer. Retrospective confidence is the subjective assessment of how correct the selected answer is (Luna et al., 2015; Goldsmith, 2016). In the current research, confidence judgments can inform us about the perceived difficulty of the questions. Even when participants predominantly select the correct answer to a given question, if the overall confidence is low, this will indicate that the question is perceived as difficult. Moreover, confidence judgements can help us detect “consensual answers” (Koriat, 2008). These types of alternatives are often confused with the correct answer due, for example, to greater familiarity with the incorrect information. For example, since the city of Sydney is so popular in terms of sports, cultural life, and being the first major city to enter the New Year, it is not uncommon for participants to choose Sydney over Canberra when questioned about the capital of Australia. In this case, the question itself is not perceived as difficult, but containing Sydney as an option is often misleading and conducive to error. Therefore, asking participants to specify their confidence in the correctness of their selections will make it possible to better characterize the questions and allow researchers to more finely tune selections in future studies based on their objectives.

Finally, in order to enable addressing different groups in such studies with more specificity, we collected data from a sample with a near-equal number of female and male participants and also report overall accuracy and confidence ratings split by gender.

## Method

### Participants

One hundred three native Russian speaking participants (all residents of the RF; mean age = 21.97, *SD* = 4.04; 58 females) recruited on social media took part in the experiment for a small monetary compensation (250 rubles per hour of experiment ~ 3.5 USD).

Participants reported a similar educational level (three participants did not report level of education completed): most of them were completing their university studies (61 in total, 42 females and 19 males), close to the 30% already had a university degree (33 in total, 14 females, 19 males), and a very small amount only had completed high school (6 in total, 2 females, 4 males). In Russia, it is compulsory to pass the Unified State Exam to get the diploma after finishing school; therefore, we can assure that all of our participants had a similar minimum of general knowledge.

We included two more additional questions about time of sleep last night and medication intake to assure the optimal state of participants to complete this long experiment. Five participants did not report the number of hours of sleep; for those who did, the mean average was *M* = 7.76 (*SD* = 1.38), and none of them reported to be under medical treatment.

### Materials and Design

Five hundred two multiple-choice GKQs were used in the experiment (see Supplementary Materials). Five hundred questions were used in the experimental part and two for pre-experimental training practice. The GKQs covered different topics—general topics (143), history (53), science (145), culture (77), and geography (82). The GKQs were selected to include all levels of difficulty (easy, medium, and difficult) by two independent university degree holders and native Russian speakers. The GKQs were retrieved from the website https://iq2u.ru/, which is directly oriented to students and professors and contains exemplars of the questions used in the Unified State Exam. These exams are compulsory in Russia either to get the high-school diploma or access to university studies (https://en.wikipedia.org/wiki/Unified_State_Exam). We also selected some other questions from https://baza-otvetov.ru/, which is a website for people interested in solving quizzes. For each question, participants had to select one alternative and rate the confidence they had in its correctness on an 11-point confidence scale ranging in 10% steps from 0% (totally unsure) to 100% (totally sure). Dependent variables were (1) accuracy and (2) confidence in the correctness of the selected answer.

### Procedure

The experiment was programmed using SR Research Experiment Builder (SR Research, Toronto, Ontario, Canada). The experiment consisted of one training session with two questions, and 10 experimental blocks with 50 questions each. The order of appearance of the questions in the experimental blocks, the blocks, as well as the placement on the screen of the alternatives was fully counterbalanced for each participant. The training questions were the same for all participants and their answers were not included into the analysis.

**Figure 1:**
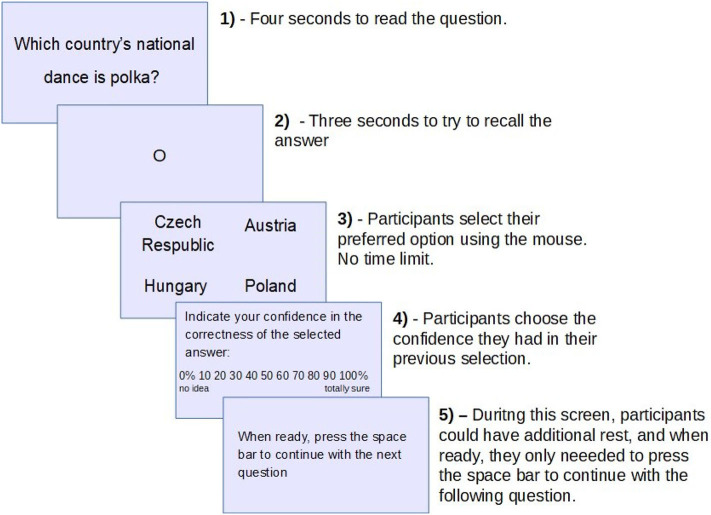
An example of one experimental trial with stages involved in answering each question.

Participants were tested individually on a computer. First, participants read and signed the informed consent form and completed the demographic data along with questions about the number of hours of sleep they got the previous night, level of education, and medication intake. They were then given instructions explaining every phase of the experiment and presented with two training questions. Then, the main experimental part started. In each trial (see Figure 1), participants first saw the question on the screen for 4 s. Then, a fixation point was presented in the middle of the screen for 3 s, during which participants were instructed to fixate and think about the answer to the question presented. This time was included in order to allow participants to retrieve potential answers. Next, four alternative answers appeared on the screen and participants had to select the one they considered correct by clicking it with the mouse. In the last step, participants selected the confidence in the correctness of their selection. The experiment lasted ~2.5–3 h. Between blocks, there were breaks of 2–3 min in which participants were instructed to move away from the computer, stretch their muscles, drink or eat small snacks, and visit the restroom if needed. Additionally, participants could also take a rest after each question.

## Results and Discussion

### General Characterization of the Questions

Out of the 500 multiple-choice GKQs, all participants consistently chose the correct answer for 8 of them. In addition, there were 7 questions more for the group of females (total of 15) and 12 for the group of males (total of 17) for which participants of each gender always selected the correct choice. There were 19 questions in the entire sample for which participants only selected one of two alternatives (the other two were never selected), and 66 questions for which one of the alternatives was never chosen. All of the questions for which one, two, or three alternatives were never selected are indicated in Supplementary Material.

### Accuracy

**Figure 2:**
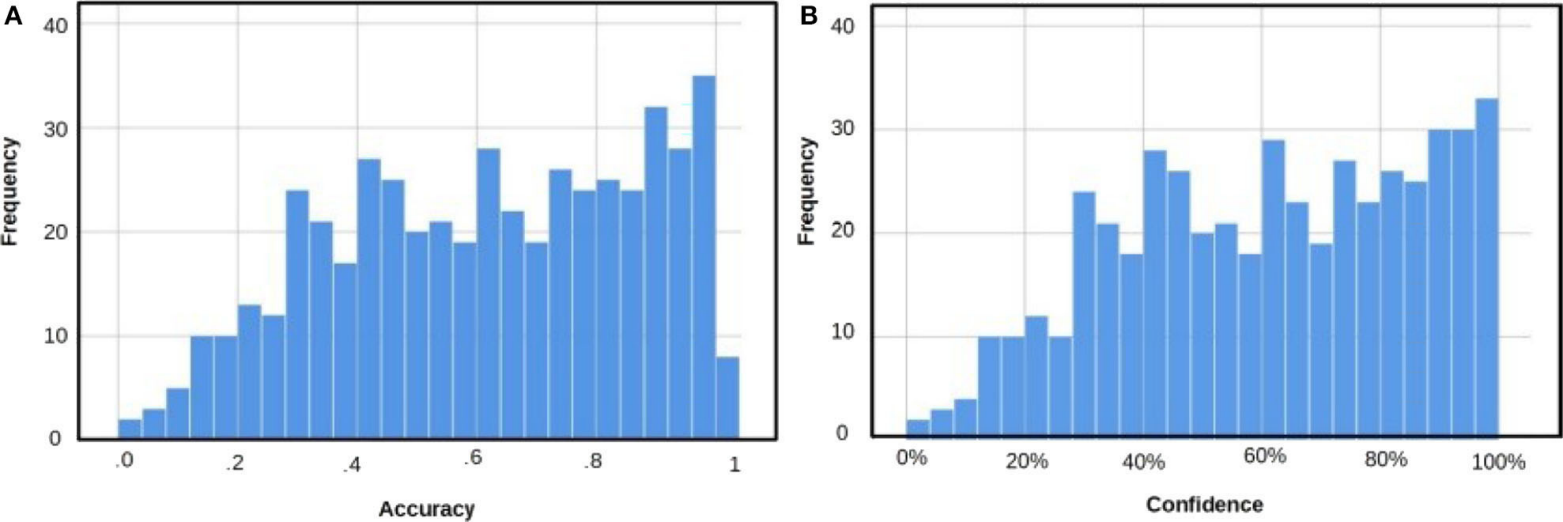
**(A)** Distribution of answers based on accuracy. **(B)** Distribution of answers based on confidence ratings.

Recognition tests are easier to solve than other memory tests such as cued recall or free recall because they are based on familiarity (Tulving, 1985; Richardson-Klavehn and Bjork, 1988; Martín-Luengo et al., 2012). This familiarity makes it challenging to create recognition questions covering all levels of difficulty. Figure 2A shows the distribution of answer accuracy. A visual inspection of Figure 2A indicates slightly more questions with accurate answers: more questions with accuracy of over 0.80 than questions with accuracy below 0.20, but overall, we obtained a homogeneous distribution. Table 1 shows the mean of accuracy split by gender for all the questions—general and for each topic. For statistical analysis, we used two-tailed independent Student *t* test, and Cohen's *d* was used to estimate effect size. There were no differences between female and male participants considering all the questions or when splitting them by topic.

**Table 1:** Mean accuracy (*SD*) split by gender (*N* female = 58; *N* male = 45) and topic with statistical analysis of between-gender differences.

**Topic**	**Gender**	**Mean accuracy (*SD*)**	**Student *t* test**	**Cohen's *d***
General	Female	0.61 (0.08)	*t*_(101)_ = −0.324, *p* = 0.747	0.064
	Male	0.60 (0.09)		
Science	Female	0.63 (0.09)	*t*_(101)_ = −0.415, *p* = 0.679	0.082
	Male	0.62 (0.09)		
History	Female	0.61 (0.09)	*t*_(101)_ = 0.710, *p* = 0.480	0.142
	Male	0.61 (0.08)		
Culture	Female	0.63 (0.10)	*t*_(101)_ = −0.128, *p* = 0.899	0.024
	Male	0.63 (0.08)		
Geography	Female	0.59 (0.10)	*t*_(101)_ = 0.157, *p* = 0.875	0.031
	Male	0.59 (0.11)		

*Bonferroni correction for multiple comparisons set the significance at p = 0.01*.

### Confidence

Figure 2B depicts the distribution of the questions based on confidence ratings without considering their accuracy. This subjective experience is important in memory tasks because it is the basis for deciding whether to keep or stop searching for the correct answer. Regardless of the accuracy, if we rate an answer with 85% confidence, we will probably stop searching for more plausible alternatives, in contrast with a confidence rating of 20% (Koriat et al., 2000). Figure 2B shows a homogeneous distribution of answers based on confidence ratings similar to the distribution of answers based on accuracy shown in Figure 2A. In this case, there are also more questions rated with high than with low confidence. This was expected considering the accuracy values and the type of memory test.

Table 2 shows the mean of confidence split by gender for all the questions—general and for each topic. As in the accuracy analysis, we calculated Student *t* test, Cohen's *d* for effect size, and Bonferroni correction for multiple comparisons when appropriate. We found that males assigned significantly higher confidence to the correctness of their answers to questions about History and Geography than females did. No other differences were found.

**Table 2:** Mean confidence (*SD*) split by gender (*N* female = 58; *N* male = 45) and topic with statistical analysis of between-gender differences.

**Topic**	**Gender**	**Mean confidence (*SD*)**	**Student *t* test**	**Cohen's *d***
General	Female	63.67 (12.91)	*t*_(101)_ = 2.013, *p* = 0.047	0.406
	Male	68.34 (9.88)		
Science	Female	64.93 (12.17)	*t*_(101)_ = 1.984, *p* = 0.050	0.398
	Male	69.39 (10.15)		
History	Female	61.06 (13.79)	*t*_(101)_ = 2.716, ***p*** **=** **0.008**	0.142
	Male	68.05 (11.77)		
Culture	Female	65.91 (11.93)	*t*_(101)_ = 2.290, *p* = 0.024	0.461
	Male	70.93 (9.75)		
Geography	Female	62.82 (12.85)	*t*_(101)_ = 2.456, ***p*** **=** **0.016**	0.494
	Male	68.62 (10.47)		

*Bonferroni correction for multiple comparisons set the significance at p = 0.01. Bold values highlight the significant differences*.

### Calibration Curves

Confidence–accuracy calibration curves show the correspondence between answer accuracy (objective measure) and confidence (subjective measure) with which answers are given in a test (Juslin et al., 1996). The graphical representation of a perfect calibration curve, where the *x*-axis represents confidence and the *y*-axis represents accuracy, is the diagonal and represents the point in which accuracy and confidence are perfectly matched (i.e., answers with 0.20 accuracy are rated with 20% confidence). There is “overconfidence” when the confidence rating is higher than the accuracy obtained (e.g., 0.50 accuracy with 70% of confidence) and “underconfidence” when the pattern is reversed, that is, lower confidence rating than the accuracy obtained (e.g., 0.50 accuracy with 20% of confidence).

**Figure 3:**
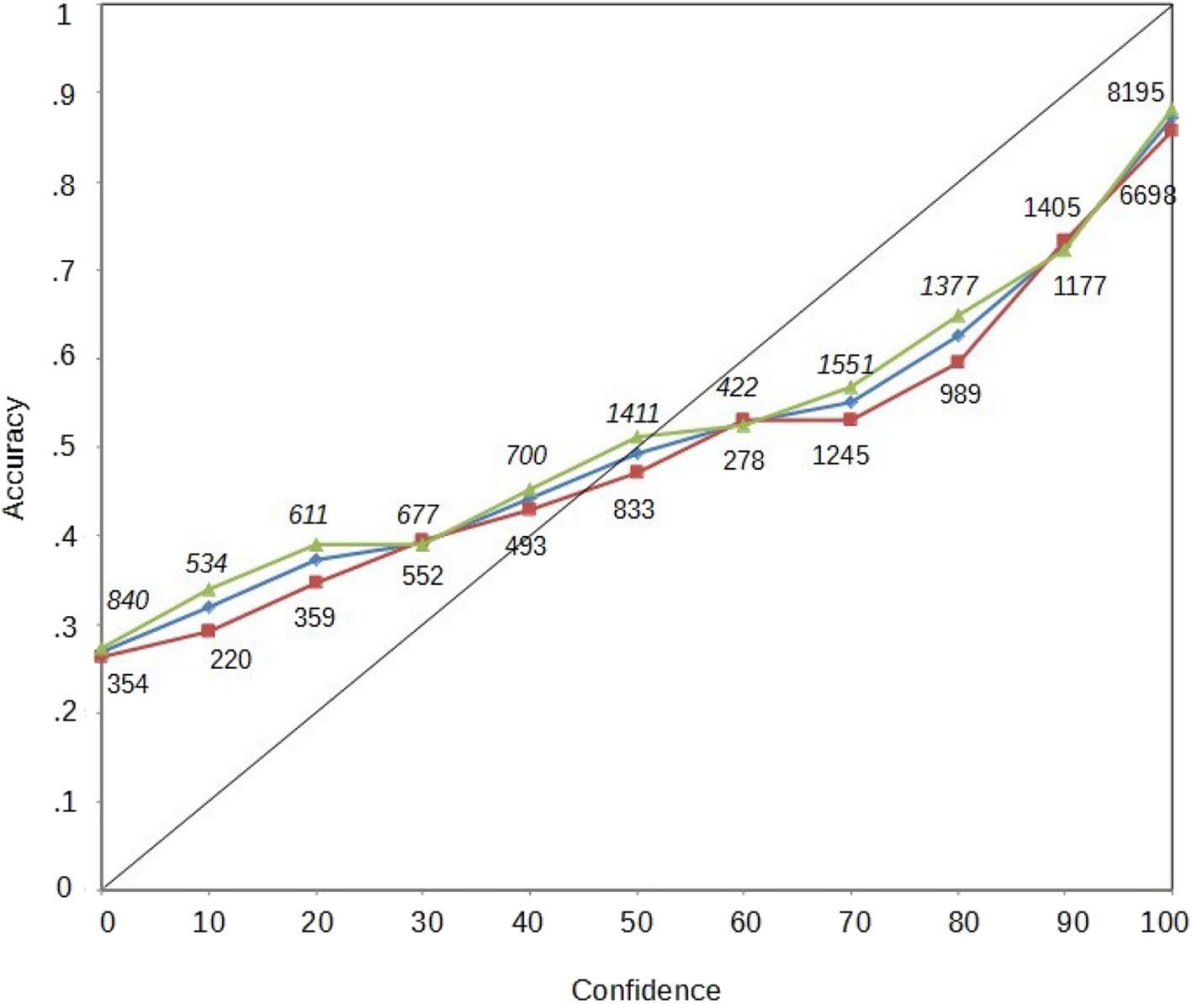
Calibration curves. Diamonds represent the calibration for the entire sample; triangles represent the calibration curve for female participants; squares represent the calibration curves for male participants. The numbers correspond to the amount of points to create the calibration.

In Figure 3, we plot three calibration curves, one for all the participants together, one with only female participants, and another one with only male participants (see Figure 3). We also provide the amount of questions used to compute each data point. For the calibration curve to be reliable, it is recommended to have 200 data points per confidence level (Juslin et al., 1996). All our points exceed that value.

The three calibration curves are similar and do not differ at any confidence level. Moreover, the three calibration curves show the so-called “hard–easy” effect (Griffin and Tversky, 1992; Luna and Martín-Luengo, 2012), which shows that we underestimate our abilities in easy tasks while overestimating them in difficult tasks. In the present case, the hard–easy effect is shown because easy questions were rated with lower confidence than they should be, and difficult questions were rated with higher confidence.

We computed the Calibration index (*C*; for calculations, see Brewer et al., 2002) to quantify the calibration curve to compare the female and male groups. A perfect calibration is indicated by 0, and higher values indicate a worse calibration. There were no differences in the calibration index between female (*M* = 0.041, *SD* = 0.027) and male (*M* = 0.040, *SD* = 0.026), *t*_(44)_ = 0.209, *p* = 0.835. Also, both *C*s were significantly different from 0: for females *t*_(44)_ = 10.156, *p* < 0.001, and for males, *t*_(44)_ = 9.950, *p* < 0.001.

## Conclusions

This study was aimed to gather norms of GKQs in Russian. As explained above, these types of studies are needed in order to better control the variables we want to manipulate. The mere translation of experimental materials from other established database (e.g., US English) disregards all cultural background, which has in fact been shown to affect problem solving (Chen et al., 2004), and culture- and language-specific materials are important to obtain objective estimates.

The multiple-choice format with four options made the present battery of questions suitable for a wide variety of experiments to be performed in Russian within a sample with similar characteristics. Moreover, the additional information obtained from the participants' subjective experience will enable experimenters to more carefully select questions to guide their experiments. All of our participants had a high-school diploma, which, for Russian citizens, means that they completed successfully the Unified State Exam, covering a range of subjects (Francesconi et al., 2019). This type of state exams to some degree ensures similar levels of general knowledge among citizens, which, in turn, implies validity of our results and applicability of our materials to populations in other regions in Russia. However, this also implies a certain limitation for the use of this battery of questions for the population who did not succeed in the Unified State Exam or did not participate in it (such as Russian speakers who are not RF residents).

Finally, in this study, we used new questions that were not previously used in other published studies. This somewhat complicated cross-cultural comparison of our results with other samples obtained in other languages and cultures. A follow-up step of this research line could be to improve this database incorporating questions used in other studies (e.g., Tauber et al., 2013; Duñabeitia et al., 2016).

## Data Availability Statement

All datasets generated for this study are included in the article/Supplementary Material.

## Ethics Statement

The studies involving human participants were reviewed and approved by Ethics committee of National Research University-Higher School of Economics. The participants provided their written informed consent to participate in this study.

## Author Contributions

BM-L, OZ, MA, and YS contributed in the design. MA collected the data. OZ and BM-L performed the data analysis. All authors contributed in writing of the manuscript.

## Open Practices Statement

The materials of this experiment can be found in the Supplementary Material, as well as the data. Raw data are available by contacting the first author.

## Conflict of Interest

The authors declare that the research was conducted in the absence of any commercial or financial relationships that could be construed as a potential conflict of interest.

## Footnotes

^1^https://w3techs.com/technologies/overview/content_language
